# Contribution of the efflux pump AcrAB-TolC to the tolerance of chlorhexidine and other biocides in *Klebsiella* spp.

**DOI:** 10.1099/jmm.0.001496

**Published:** 2022-03-24

**Authors:** Matthew E. Wand, Elizabeth M. Darby, Jessica M. A. Blair, J. Mark Sutton

**Affiliations:** ^1^​ UK Health Security Agency, Research and Development, Porton Down, Salisbury, Wiltshire, SP4 0JG, UK; ^2^​ Institute of Microbiology and Infection, College of Medical and Dental Sciences, University of Birmingham, Birmingham, UK

**Keywords:** chlorhexidine, *acrAB*-TolC, *ramR*, *smvAR*, cationic biocide, *Klebsiella*

## Abstract

**Introduction.** We are becoming increasingly reliant on the effectiveness of biocides to combat the spread of Gram-negative multi-drug-resistant (MDR) pathogens, including *Klebsiella pneumoniae *. It has been shown that chlorhexidine exposure can lead to mutations in the efflux pump repressor regulators SmvR and RamR, but the contribution of each individual efflux pump to biocide tolerance is unknown.

**Hypothesis.** Multiple efflux pumps, including SmvA and AcrAB-TolC, are involved in increased tolerance to biocides. However, strains with upregulated AcrAB-TolC caused by biocide exposure are more problematic due to their increased MDR phenotype.

**Aim.** To investigate the role of AcrAB-TolC in the tolerance to several biocides, including chlorhexidine, and the potential threat of cross-resistance to antibiotics through increased expression of this efflux pump.

**Methodology.** Antimicrobial susceptibility testing was performed on *K. pneumoniae* isolates with *ramR* mutations selected for after exposure to chlorhexidine, as well as transposon mutants in components and regulators of AcrAB-TolC. RTPCR was used to detect the expression levels of this pump after biocide exposure. Strains from the globally important ST258 clade were compared for genetic differences in *acrAB*-TolC and its regulators and for phenotypic differences in antimicrobial susceptibility.

**Results.** Cross-resistance to antimicrobials was observed following mutations in *ramR*. Exposure to chlorhexidine led to increased expression of *acrA* and its activator *ramA*, and transposon mutants in AcrAB-TolC have increased susceptibility to several biocides, including chlorhexidine. Variations in *ramR* within the ST258 clade led to an increase in tolerance to certain biocides, although this was strain dependent. One strain, MKP103, that had increased levels of biocide tolerance showed a unique mutation in *ramR* that was reflected in enhanced expression of *acrA* and *ramA*. MKP103 transposon variants were able to further enhance their tolerance to specific biocides with mutations affecting SmvA.

**Conclusions.** Biocide tolerance in *K. pneumoniae* is dependent upon several components, with increased efflux through AcrAB-TolC being an important one.

## Introduction

Infection prevention is critical to combat the rise of multi-drug-resistant (MDR) bacterial infections. One component of infection prevention is the use of disinfectants and antiseptics (biocides) to prevent the colonization and transmission of pathogens. There is, however, concern that the increase in use of these biocides could lead to increased bacterial tolerance to biocides and/or cross-resistance to frontline antibiotics. This is because many potential biocide resistance mechanisms, such as upregulation of specific bacterial efflux pumps, are common antibiotic resistance determinants, and biocide tolerance genes are carried on antimicrobial resistance (AMR) plasmids [1]. When both *Salmonella enterica* and *Stenotrophomonas maltophilia* were exposed to the biocide triclosan, increased resistance to certain antibiotics was shown through upregulation of particular efflux pumps [2, 3]. In Gram-negative bacteria probably the most clinically relevant efflux pumps are members of the resistance–nodulation–division (RND) family, which recognize a broad range of substrates, including antibiotics and biocides [4]. This family includes the well-characterized members MexAB-OprM from *Pseudomonas aeruginosa *, AdeABC in *Acinetobacter baumannii* and the *Enterobacteriaceae* MDR efflux pump AcrAB-TolC [5–7].

AcrAB-TolC is a tripartite RND efflux pump comprising an outer membrane channel TolC, the inner membrane transporter AcrB and the periplasmic membrane fusion protein AcrA. In *Escherichia coli* the expression of *acrAB* is primarily controlled by MarA [8, 9], with *marA* overexpression generating increased resistance to multiple antibiotics, disinfectant pine oils and triclosan [10, 11], but in *S. enterica* and *Klebsiella pneumoniae* the major regulator is RamA [12]. The pump is also regulated by a variety of other different factors, including SoxS and the TetR family transcriptional repressors, AcrR and EnvR.

*K. pneumoniae* is an important opportunistic pathogen that is prominent in causing respiratory and urinary tract infections. A few high-risk sequence types (STs), e.g. ST258, ST11, ST512, ST14 and ST101, are responsible for the global dissemination of carbapenemases and other multi-drug resistance determinants [13–15]. In *K. pneumoniae* several efflux pumps have been linked to tolerance to various biocides, including MdfA, MdtK and AceI [16]. For chlorhexidine, increased tolerance was shown in strains with an upregulated major facilitator superfamily (MFS) efflux pump SmvA, which was due to mutations in the adjacent repressor (SmvR) [17]. Strains lacking SmvA also have increased chlorhexidine susceptibility and SmvA has additionally been linked to tolerance in *Klebsiella* to another cationic biocide, octenidine [18]. Other potential mechanisms of increased chlorhexidine tolerance include efflux pumps such as CepA (FieF) [19, 20] and EmrAB (KpnGH) [21]. Efflux is linked to increased biocide tolerance and these strains often have upregulated MDR efflux pumps, such as AcrAB-TolC. However, little is known about the contribution of these individual efflux pumps to biocide tolerance. Mutations and insertions/deletions in *ramR* (the repressor of RamA) have been shown to increase the expression of *ramA* by preventing RamR binding to the *ramA* promoter, which caused an increase in the expression of *acrAB-TolC* in *K. pneumoniae* [22, 23], resulting in an MDR phenotype [24]. Exposure to triclosan and benzalkonium chloride in *K. pneumoniae* frequently yielded *ramA*-overexpressing mutants [25], whilst mutations in RamR were identified in *S. enterica* following exposure to several biocides [26]. Therefore, multiple efflux pumps may contribute towards increased biocide tolerance.

The aim of these experiments was to study the relative contribution of AcrAB-TolC to the tolerance to chlorhexidine and other biocides in *Klebsiella* using already generated chlorhexidine adapted mutants to understand which efflux pump is the primary resistance mechanism. We utilized SmvAR as a comparator since we have previously shown that this pump is important for chlorhexidine tolerance but contributes little to antibiotic resistance. Therefore, changes in AcrAB-TolC are potentially more clinically relevant due to an associated increase in antibiotic resistance. Strains with upregulated *acrAB*-tolC are also isolated more regularly in the clinic.

This study shows that AcrAB-TolC has an important role in the tolerance of many biocides and that specific changes in the sequence of the main regulators of AcrAB-TolC, *ramAR* and *acrR* affect susceptibility to chlorhexidine and other cationic biocides. Although the study mainly focuses on AcrAB-TolC, we cannot ignore the importance of other efflux pumps, namely SmvAR. This study shows that the response to biocides in *Klebsiella* is multifaceted and the contribution of each individual efflux pump is likely to be biocide and strain dependent.

## Methods

### Bacterial strains and culture conditions

All *Klebsiella* strains were grown in tryptic soy broth (TSB) with aeration or on tryptic soy agar at 37 °C unless stated otherwise. The strains chosen include a mixture of clinical isolates, primarily isolated post-2015, from the UK. The majority are carbapenem-resistant *K. pneumoniae* ST258 isolates. Whole-genome sequences were available for all strains used in this study. Important strain characteristics, including antibiotic resistance profiles generated by RES-FINDER, are listed in Table S1 (available in the online version of this article). Transposon mutants from *K. pneumoniae* MKP103 are also listed in Table S3 and were described previously [27]. Transposon mutants KP02744 (MKP103∆*acrA*), KP02740 (∆*acrB*), KP02746 (∆*acrR*), KP03203 (∆*ramA*) and KP03197 (∆*ramR*) were whole-genome sequenced and mapped against the parental MKP103 strain to confirm that the only mutations that were found in each strain were the respective transposon insertions. *K. pneumoniae* transposon mutants were adapted to chlorhexidine using a stepwise method. Cultures were initially grown in subinhibitory concentrations of chlorhexidine (one-quarter the MIC level) and then every 2 days passaged into double the previous chlorhexidine concentration until a concentration of eight times the initial MIC was reached. Cultures were subsequently passaged on agar plates 10 times in the absence of chlorhexidine selection. Ethidium bromide dye uptake assays were performed as previously described [28]. Briefly, strains were cultured to mid-log phase (OD_600_ 0.4) in the presence of sub-MIC chlorhexidine levels (8 and 4 mg l^−1^) in TSB. Cells were then pelleted at 3500 r.p.m. for 10 min and resuspended in 0.02 M potassium phosphate buffer (pH 7.0) with 1 mM MgCl_2_. Cells were adjusted to OD_600_ 0.2 and 190 µl was loaded into a flat-bottomed, black, 96-well plate (Greiner Bio-one, Stonehouse, UK). Subsequently, 10 µl of ethidium bromide (50 mg l^−1^) was added to each well. The accumulation of ethidium bromide was quantified using a FLUOstar Omega plate reader (BMG Labtech) where fluorescence was measured using excitation and emission filters at 544 and 590 nm, respectively, and a gain multiplier of 1460 for 2 h.

### Whole-genome sequencing (WGS)

*K. pneumoniae* genomic DNA was prepared using a Wizard Genomic DNA purification kit (Promega). Whole-genome sequencing of chlorhexidine-exposed isolates was performed by PHE-GSDU (Public Health England Genomic Services and Development Unit) on an Illumina HiSeq 2500 with paired-end read lengths of 150 bp. All sequencing analyses were performed using PHE Galaxy [29]. FastQ files were quality trimmed using Trimmomatic and reads from chlorhexidine-exposed isolates were mapped to their respective pre-exposure chromosomal sequence using BWA0.7.5. Bam files were generated using Samtools and VCF files were constructed using GATK2 Unified Genotyper 0.0.7. They were further filtered to identify high-confidence SNPs using the following criteria: mapping quality, >30; genotype quality, >40; variant ratio, >0.9; read depth, >10. BAM files were visualized in Integrative Genomics Viewer (IGV) version 2.3.55 (Broad Institute). All sequences have been deposited with the National Center for Biotechnology Information (NCBI) under the Bioproject ID PRJNA777533.

### Determination of MIC and MBC

The minimum inhibitory concentration (MIC) and the minimum bactericidal concentration (MBC) of various antibiotics and disinfectants/antiseptics for bacterial isolates were determined using a standard broth microdilution method detailed by the European Committee on Antimicrobial Susceptibility Testing (EUCAST) with a starting inoculum of 1×10^5^ c.f.u. ml^−1^, except that 96-well polypropylene plates (Griener Bio-One Ltd, Stonehouse, UK) were used instead of polystyrene plates to test colistin. The optical density at 600 nm (OD_600_) was measured after 20 h of static incubation at 37 °C, and the MIC was defined as the lowest concentration of antibiotic/disinfectant at which no bacterial growth was observed. MBCs were measured by plating out onto TSA plates 10 µl of MIC dilutions from and including the MIC level and the subsequent three further higher biocide concentrations (where applicable). The efflux pump inhibitors phenylalanine-arginine β-naphthylamide (PaβN) and carbonyl cyanide 3-chlorophenylhydrazone (CCCP) were added at concentrations of 25 and 10 mg l^−1^ respectively.

### Real-time PCR

Overnight cultures grown in TSB were back-diluted to an OD_600_ of 0.1 in TSB and grown for a further 1 h. Cultures were back-diluted to an OD_600_ of 0.25 in TSB alone (unexposed) or TSB containing sub-MIC (8 mg l^−1^) or lethal (128 mg l^−1^) concentrations of chlorhexidine and incubated for 30 min with shaking at 37 °C. Cultures were then harvested using RNA protect (Qiagen) and RNA was extracted using the RNeasy minikit (Qiagen) according to the manufacturer’s instructions. cDNA was synthesized, and real-time PCR was carried out and analysed as previously described [17] using *K. pneumoniae infB*, *gapA* and *rpoB* as internal control genes. Primers used have already been described [17] except for *acrA* primers (KP*acrA*665R3 TCATTGCTCGACTGGGTGAC; KP*acrA*586F3 CAGAATGGTCAAACGACCGC) and *ramA* primers (KP*ramA*332R3 CGACTGTGGTTCTCTTTGCG; KP *ramA* 260F3 AGACCTTTACCCGCGTCTTC).

### Statistical analysis

Real-time PCR data were analysed for significance using Student’s unpaired *t*-test. For significance, *P* values <0.0001 ****, 0.001–0.0001 ***, 0.01–0.001 **, 0.05–0.01 * and ≥0.05 non-significant were used.

## Results and discussion

### Chlorhexidine-adapted *Klebsiella* strains with RamR mutations have increased resistance to several antibiotics

Chlorhexidine-exposed *Klebsiella* strains had shown mutations in *ramR* previously [18] and to understand the contribution of AcrAB-TolC, these mutants were analysed for their change in antibiotic and biocide tolerance. Strain 6 CHD [which contains mutations in both *smvR* (Del nucleotides 48–54) and *ramR* (E7STOP)] and *Klebsiella oxytoca* strain CFI_080_KPC2 CHD (which contains a deletion in *ramR*) both showed decreased susceptibility to several antibiotics and biocides (Table 1a and b). Many of these antibiotics are known substrates for *acrAB-TolC*, such as the fluoroquinolones and doxycycline. SmvAR has been shown to have a negligible effect on antibiotic susceptibility and is more likely a selective pump for certain cationic compounds [18]. Therefore, the change in antibiotic MICs observed in strain 6 CHD are likely to be because of mutations in *ramR*. For certain biocides (Table 1b) strain 6 CHD showed a larger fold increase in biocide resistance – e.g. CPC, HDPCM and cetrimide (up to 16-fold) – than CFI_080_KPC2 CHD (2–8-fold) when compared to their respective wild-types. This potentially indicates that the presence of mutations in regulators of both AcrAB-TolC and SmvA has a cumulative effect on tolerance to these biocides and that they are substrates for both pumps.

**Table 1.** Susceptibility of chlorhexidine-adapted *Klebsiella* strains to various antibiotics (a) and biocides (b)

(a)															
	CIP	LVX	NOR	MXF	NAL	FOX	CAZ	CTX	AZM	DOX	CHL	GEN	TOB	CST	TGC
6 WT	0.06	0.125	0.25	0.5	16	4	0.25	0.06	32	4	8	2	4	0.5	1
6 CHD	**0.5**	**1**	**2**	**2**	**64**	**64**	**2**	**0.5**	**>64**	**32**	**64**	2	4	0.5	**2–4**
CFI_080_KPC2 WT	0.03	0.125	0.25	0.25	8	64	>64	>64	64	8–16	4	8	16	0.5	1
CFI_80_KPC2 CHD	**0.125**	**0.5**	**1**	**1**	**16**	>64	>64	>64	>64	**32**	**16**	8	8–16	0.5	**2–4**
**(b)**															
	**ALX**	**CET**	**DQC**	**TRC**	**CPC**	**HDPCM**	**CHD**	**CTAB**	**DDAB**	**OCT**	**BAC**	**BEC**	**Eth**		
6 WT	4	0.0009	256	0.25	4	4	32	16	8	4	8	32	6.25		
6 CHD	4	**0.007**	256	**1**	**64**	**64**	**128**	**128**	**16**	4	**32**	**64**	6.25		
CFI_80_ KPC2 WT	2	0.003	128	0.125	8	8	16	16	8	4	16	32	6.25		
CFI_80_ KPC2 CHD	2	**0.03**	128	**1**	**16**	**16**	**64**	**128**	**16**	4	16	32	6.25		

MIC values (mg l^−1^) except where indicated are shown for the antibiotics ciprofloxacin (CIP), levofloxacin (LVX), norfloxacin (NOR), moxifloxacin (MXF), naladixic acid (NAL), cefoxatin (FOX), ceftazidime (CAZ), cefotaxime (CTX), aztreonam (AZM), doxycycline (DOX), chloramphenicol (CHL), tobramycin (TOB), colistin (CST) and tigecycline (TGC) (1A), and for the biocides alexidine dihydrochloride (ALX), cetrimide (CET) (%), dequalinium chloride hydrate (DQC), triclosan (TRC), cetylpyridinium chloride (CPC), hexadecylpyridinium chloride monohydrate (HDPCM), chlorhexidine digluconate (CHD), cetyltrimethylammonium bromide (CTAB), didecyldimethylammonium bromide (DDAB), octenidine hydrochloride (OCT), benzalkonium chloride (BAC), benzethonium chloride (BEC) and ethanol (Eth) (%). Values in bold indicate an increase of ≥fourfold in MIC levels for the chlorhexidine adapted mutants (strain CHD) versus the wild-type (WT).

### Exposure to chlorhexidine causes upregulation of *acrAB*

Early log-phase *K. pneumoniae* strain MGH 78578 was exposed to sub-lethal and lethal levels of the cationic biocides chlorhexidine and octenidine. This included a biocide (chlorhexidine) where an increase in MIC was observed in strains containing *ramR* mutations following chlorhexidine adaptation, and a biocide (octenidine) where no increase in MIC was observed. Increased expression of *acrA* (approximately 2.7-fold for sub-lethal and 2-fold for lethal concentrations of both biocides) and its regulator *ramA* (14.7-fold for sub-lethal and 4.7-fold for lethal concentrations of both biocides) was shown (Fig. S1). This showed that *Klebsiella* responds to the presence of both sub-lethal and lethal concentrations of chlorhexidine and octenidine through increased expression of *acrAB-TolC*, but that these increased expression levels do not necessarily correlate with a change in MIC. The changes in the expression levels of *acrA* and *ramA* was not as large a fold increase as shown for *smvA* [18], but this may be due to a lower *smvA* basal level.

Since exposure to sub-lethal concentrations of chlorhexidine led to higher transcript levels of *acrAB-TolC*, it was hypothesized that this increased expression could lead to elevated MICs for several antibiotics. *K. pneumoniae* strains were challenged with antibiotics known to be substrates of the AcrAB-TolC pumps in the presence of sub-lethal levels of chlorhexidine (4 and 8 mg l^−1^). However, the results showed that the effect of chlorhexidine with the antimicrobial was additive; the presence of chlorhexidine led to decreased MIC values for all antibiotics tested, which were further decreased as the chlorhexidine concentration increased (Table S2). This showed that chlorhexidine is working in synergy with the antibiotics, probably through permeation of the bacterial membrane, an observation previously described with another cationic biocide alexidine [30]. Experiments using fluorescent dyes, which are often used to study efflux, showed increased accumulation of the dye in the presence of sub-inhibitory concentrations of chlorhexidine (data not shown). The presence of sub-lethal levels of chlorhexidine is likely to induce an increased stress response, which possibly masks the effect of upregulation in *acrAB-tolC* through *ramA*. It has been shown that the rate of induction of *acrAB* is dependent upon the rate of stress introduction [31]. However, in *Salmonella* the expression of *ramA* was found to be unchanged when bacteria were challenged with several antibiotic substrates of AcrAB-tolC [32]. Therefore, the response of *ramA* and *acrA* after challenge with cationic biocides is condition and species specific but does not necessarily indicate a role in chlorhexidine tolerance.

### Transposon mutants in *acrAB* show increased susceptibility to biocides

One strain in our collection, NCTC 7427, is an ST86 strain and contains a premature stop codon in *acrB* and thus an inactive AcrAB-TolC pump. Comparison with the only other ST86 strain in our collection, KPUK02, showed that susceptibility to several biocides, including chlorhexidine, triclosan, CTAB and benzalkonium chloride was increased (≥fourfold) in NCTC 7427 (Table 2). This shows that for ST86 strains AcrAB-TolC is an important component for tolerance to several biocides, such as chlorhexidine, benzalkonium chloride and triclosan, but not for others, e.g. silver nitrate, glutaraldehyde and sodium hypochlorite. Comparison of the genomes of NCTC 7427 and KPUK02 showed that NCTC 7427 contained identical DNA sequences for all the other major efflux pumps, such as *oqxAB*, *emrAB*, *cepA* and *smvA*, and their regulators, again suggesting that the changes seen are due to the presence/absence of AcrAB. The only exception is that NCTC 7427 lacked a homologue to KpnEF, which is thought to have some activity against certain biocides [21].

**Table 2.** Comparison of ST86 strains, NCTC 7427 that lacks a functional AcrAB-TolC efflux pump and KPUK02 that has an intact AcrAB-TolC efflux pump. Values in bold indicate an increase of ≥fourfold in MIC/MBC value when KPUK02 is compared to NCTC 7427. All values given as mg l^−1^ unless stated otherwise

	NCTC 7427		KPUK02	
	MIC	MBC	MIC	MBC
Triclosan (TRC)	≤0.06	>2	**0.25–0.5**	>2
Octenidine (OCT)	1	1	2	2
Dequalinium chloride (DQC)	>512	>512	>512	>512
Bile salts	>512	>512	>512	>512
Benzethonium chloride (BEC)	8	8	**32**	**32**
Silver nitrate (AgNO_3_)	32	32–64	64	64
Hexadecylpyridinium chloride monohydrate (HDPCM)	2	2	2–4	4
Glutaraldehyde	0.19%	0.19%	0.19%	0.19%
Acriflavine	32	32	**>512**	**>512**
Hexadecyltrimethylammonium bromide (CTAB)	2	2	**8**	**8**
Benzalkonium chloride (BAC)	4–8	8	**32**	**64**
Didecyldimethylammonium bromide (DDAB)	2	2	**4–8**	**8**
Chlorhexidine digluconate (CHD)	2	2	**16**	**16**
Cetrimide (CET)	≤0.0003 %	0.0015%	**0.0015%**	0.0015%
Ethanol	3.13%	6.25%	3.13–6.25 %	3.13–6.25 %
Sodium hypochlorite	156 p.p.m.	312 p.p.m.	156–312 p.p.m.	312 p.p.m.
Crystal violet	0.000125%	0.000125%	**>**0.004 %	**>**0.004 %
Cetylpyridinium chloride (CPC)	2	2	4	4–8
Peracetic acid	0.08%	0.08%	0.08%	0.08%
Alexidine dihydrochloride (ALX)	1–2	1–2	2	2
Hydrogen peroxide	0.03%	0.03%	0.03%	0.03%

Transposon mutants in *acrAB* and specific regulators known to affect *acrAB* expression in *K. pneumoniae* strain ST258 strain MKP103 were also analysed for their tolerance to several biocides (Table S3). Mutants in the efflux pump *acrAB* reduced the MIC for chlorhexidine (8–16-fold), DQC (8-fold), triclosan (2–4-fold) and several others (2-fold). This agrees with the results generated from the ST86 strains. However, the removal of the regulators *ramR* and *acrR* did not alter the MIC/MBC values except for CTAB. The antibiotic MICs against the same set of mutants also showed similar results, with the loss of the components of the pump having more of an effect than loss of the regulators (Table S4). Regulators such as RamR and AcrR act as repressors where mutations in these genes often result in loss of function. Therefore, strains with mutations in RamR and AcrR will have upregulated efflux. Subsequent removal of *ramR* and/or *acrR* from these strains is likely to have little noticeable effect on the MIC/MBC values. However, removal of the efflux pump itself will have a greater effect since the bacterium is moving from a state of upregulated efflux to a state of no efflux.

### Sequence variation within RamR and AcrR leads to increased expression of *acrAB* and may contribute towards increased biocide tolerance

The genome sequence of MKP103 was analysed for a possible explanation for the constant *acrAB* upregulation. This revealed a unique amino acid change (G42V) in RamR relative to other ST258 strains in our collection. It is well known that changes in RamR regulate the expression levels of *acrAB* [33–35]. Therefore, it is plausible that this change in strain MKP103 leads to constant upregulation of *acrAB*, and removal of the repressors *ramR* and *acrR* would have minimal effect on antimicrobial tolerance. Sequence analysis of all ST258 strains in our collection showed other strains with unique changes in RamR and RamA but not in other potential AcrAB-TolC regulators.

Six ST258 strains were analysed for their susceptibility to biocides. Strain MKP103 had increased MICs (often fourfold) to DDAB, chlorhexidine, HDPCM, triclosan and cetrimide when compared to the other strains (Table 3). This increase in tolerance could be due to elevated expression of *acrAB-TolC*. To investigate this the basal expression levels of *acrA* and *ramA* for all ST258 strains were measured. For comparison and due to their importance in biocide tolerance, the levels of *smvAR* were also investigated, although there were no sequence differences for these genes within the ST258 strains. The results showed that MKP103 did indeed have elevated expression levels of *acrA* and *ramA* relative to other ST258 strains (Table 4). Although not to the same level as MKP103, strain CFI_147_KPC-2 also had elevated *acrA* and *ramA* expression. This strain contains the unique mutation L54F in RamR and shows that both mutations in RamR (G42V and L54F) cause derepression of *ramA* that in turn leads to increased expression of *acrAB-TolC*. However, only in MKP103 does this change result in elevated MIC values for biocides. For *smvAR* expression levels, no significant difference was observed between all strains.

**Table 3.** Biocide tolerance for *K. pneumoniae* strains from sequence type 258 (ST258) and their genetic differences in the AcrAB regulators *ramAR* and *acrR*

Strain	ALX	DDAB	BEC	BAC	CHD	DQC	OCT	HDPCM	TRC	Eth	CET	CPC	CTAB	RamR	RamA	AcrR
ST258																
NCTC 13438	4	4	32	16	32	256	2–4	8	1	6.25	0.0015	16	32	A37V	N18Y	–
46704	4	4	32	16	16	256	4	8	0.5	6.25	0.0015	16	32	–	–	–
CFI_131_KPC2	2	2	32	8	16	256	4	4	1	3.125	0.0007	8	16	Absent	Absent	–
CFI_141_KPC3	4	4	32	16	16	256	2	8	0.5	3.125	0.003	16	32	–	–	–
CFI_147_KPC2	2	2	64	16	16	256	2	4	0.5	3.125	0.0007	8	8	L54F	–	–
MKP103	4	8–16	32	16–32	64–128	256	4	16	4–8	6.25	0.003–0.007	16	32–64	G42V	–	–

MIC values (mg l^−1^) except where indicated are shown for the biocides alexidine dihydrochloride (ALX), didecyldimethylammonium bromide (DDAB), benzethonium chloride (BEC), benzalkonium chloride (BAC), chlorhexidine digluconate (CHD), dequalinium chloride hydrate (DQC), octenidine hydrochloride (OCT), hexadecylpyridinium chloride monohydrate (HDPCM), triclosan (TRC), ethanol (Eth) (%), cetrimide (CET) (%), cetylpyridinium chloride (CPC) and cetyltrimethylammonium bromide (CTAB). For other genes that have been implicated as regulators of AcrAB-TolC, including *marAR*, *soxRS*, *rob*, *sdiA*, *fis* and *envR*, the sequence for all ST258 strains was identical except for a premature stop codon in *soxR* (Q97STOP) in strain CFI_141_KPC3.

**Table 4.** Expression levels (fold change) for genes in *K. pneumoniae* ST258 strains relative to strain 46704. Significance is indicated

	*acrA*	*ramA*	*smvA*	*smvR*
NCTC 13438	0.691	3.204	1.067	0.542
46704	1	1	1	1
CFI_131_KPC2	0.427	0	0.4	0.734
CFI_141_KPC3	0.912	0.733	0.492	0.655
CFI_147_KPC2	4.639*	15.554**	1.514	1.109
MKP103	8.159*	20.329**	1.143	1.385

Since the rate of efflux does not always correlate with baseline MIC/MBC values [36, 37], we attempted to measure the impact of chlorhexidine-mediated *acrAB-tolC* expression on ethidium bromide dye accumulation after challenge with chlorhexidine for the ST258 *K. pneumoniae* strains. Unfortunately, despite repeated attempts, we were unable to gain reproducible data, probably because chlorhexidine acts as a membrane permeabilizer.

### Chlorhexidine and other biocide tolerance levels in *Klebsiella* are dependent on multiple efflux pumps

To further attempt to decipher the importance of the individual efflux pumps SmvA and AcrAB-TolC, MKP103 transposon mutants in *ramA*, *ramR* and *smvA* were adapted to chlorhexidine in a stepwise manner. This was to generate strains that had different levels of expression of *acrAB-TolC* and *smvA* efflux pumps. Adaptation of KP02744 (∆*acrA*) and KP02740 (∆*acrB*) to chlorhexidine was attempted, but despite repeated efforts we were unsuccessful. Exposure of strains KP03202 (∆*ramA*), KP03197 (∆*ramR*) and the parental MKP103 to chlorhexidine selected for mutations in *smvR*, but no mutations in *acrAB* or its regulators were detected in KP05925 (∆*smvA*) (Table S5). This is probably due to the already upregulated *acrAB-TolC* expression levels, meaning that additional mutations would have minimal effect. Comparison of the MIC values for various biocides for the chlorhexidine adapted mutations concluded that again a cumulative effect was seen, particularly for chlorhexidine (fourfold increase) in strains that had mutations in *smvR* and already upregulated *acrAB-TolC* (Table S6). Strain MKP103∆*smvA* CHD showed no increase in biocide MIC values, except for chlorhexidine (two–fourfold). That it was not possible to generate adapted mutants in strains KP02744 (∆*acrA*) and KP02740 (∆*acrB*) supports an important role for AcrAB-TolC in the export of chlorhexidine in MKP103.

To further aim to separate the role of AcrAB-TolC and SmvA in biocide tolerance, the efflux pump inhibitors PaβN and CCCP were employed on selected *Klebsiella* strains, including those with upregulated *acrAB-TolC*. PaβN is a competitive inhibitor of AcrAB-TolC [38] but should not affect the MFS pump SmvA. CCCP has been shown to enhance the efficacy of chlorhexidine as well as colistin in *Klebsiella* [17] and is an uncoupler of the proton motive force. This should theoretically affect both RND and MFS pumps, but will also have pleiotropic effects on other aspects of membrane function [39]. The addition of CCCP was only effective for chlorhexidine, whilst the presence of PaβN resulted in reduced MIC values for CET, CPC, HDPCM and CTAB, as well as the antibiotics CHL and CIP, which are known to be subject to efflux by AcrAB-TolC (Table 5). This indicated that AcrAB-TolC is a major efflux pump in *Klebsiella* for those biocides, but that its role in efflux of chlorhexidine is either fully complemented by SmvA or it is not inhibited by PaβN. PaβN acts as a competitive inhibitor in competition with antimicrobials in the AcrAB-TolC binding pocket [40] and therefore the binding affinity for chlorhexidine to AcrAB-TolC may be higher relative to PaβN and thus chlorhexidine is able to outcompete PaβN, rendering it ineffective. Chlorhexidine and PaβN may also interact with different amino acids in AcrAB. This has been shown for PaβN and tetracycline, where the binding pockets for each chemical do not overlap [41]. Studies have shown that the addition of CCCP did not affect sensitivity to carbapenems or tigecycline in *Enterobacteriaceae* [42] but these antibiotics have been shown to subject to efflux by AcrAB-TolC in *Klebsiella* [43, 44]. Having previously shown that SmvA is an important efflux pump for several cationic biocides in *Klebsiella* [18], it was perhaps surprising that the addition of CCCP had no effect on susceptibility to the biocides tested. One solution is that CCCP has no effect on SmvA, and that the effect with chlorhexidine is nothing to do with SmvA. Another well-studied MFS efflux pump, EmrAB, has been shown to efflux CCCP [45], and therefore the major effect of CCCP might instead be to act directly on the cell membrane, which is the site of action for chlorhexidine. This potentially shows that chlorhexidine has a different mechanism of action from other cationic biocides, resulting in different resistance mechanisms.

**Table 5.** The effect of the efflux pump inhibitors PaβN and CCCP on biocide susceptibility in chlorhexidine-adapted strains and their respective wild-types. Numbers highlighted in bold indicate a ≥fourfold change in MIC relative to no EPI (alone)

	CHD			CET			CPC			HDPCM		
	Alone	+PaβN	+CCCP	Alone	+PaβN	+CCCP	Alone	+PaβN	+CCCP	Alone	+PaβN	+CCCP
6	8–16	8–16	**1**	0.0007	**≤0.0003**	0.0007	4	4	16	4	2	4
6 CHD	64	64	**2**	0.007	**≤0.0003**	0.015	32	**4**	64	64	**4**	64
CFI_080_KPC2	4–8	8–16	**1**	0.0007	**≤0.0003**	0.007	8	**2**	**32**	16	**2**	32
CFI_080_KPC2 CHD	16	16	**2**	0.007	**≤0.0003**	0.015	32	**2**	64	16	**2**	**64**
MKP103	128	128	**1**	0.007	**≤0.0003**	0.015	32	**4**	64	16	**4**	**64**
	**CTAB**			**CHL**			**CIP**					
	Alone	+PaβN	+CCCP	Alone	+PaβN	+CCCP	Alone	+PaβN	+CCCP			
6	8	**2**	32	8	**2**	16	0.25	**≤0.06**	0.125			
6 CHD	32	**8**	64	64	**8**	32	0.25	0.125	0.25			
CFI_080_KPC2	16	**4**	32	8	**2**	16	≤0.06	≤0.06	≤0.06			
CFI_080_KPC2 CHD	64	**4**	128	16	**4**	16	≤0.06	≤0.06	≤0.06			
MKP103	32	**8**	128	128	**8**	64	>64	>64	>64			

Antimicrobials tested included chlorhexidine digluconate (CHD), (%), cetrimide (CET), cetylpyridinium chloride (CPC), hexadecylpyridinium chloride monohydrate (HDPCM), cetyltrimethylammonium bromide (CTAB), chloramphenicol (CHL and ciprofloxacin (CIP). All values in mg l^−1^ except for CET whose values represent % of active ingredient.

## Conclusion

This study provides evidence that AcrAB-TolC is an important efflux pump in *K. pneumoniae* for specific biocides. Adaption to chlorhexidine, although predominantly driven by mutations in *smvAR*, can result in mutations in the *acrAB* regulator *ramR*, which leads to decreased susceptibility to several antibiotics. This study shows an important role for both SmvA and AcrAB-TolC in the efflux of biocides in *Klebsiella *, with each pump likely to efflux multiple biocides, including chlorhexidine. Exposure to chlorhexidine can result in a decrease in susceptibility to many antibiotics through *ramR* mutations and, therefore, whilst adaptation to chlorhexidine is more likely to result in changes to SmvAR in a laboratory setting, within the clinic, strains with *ramR* mutations are more problematic due to potential cross-resistance to antibiotics.

## Supplementary Data

Supplementary material 1
