# Supplementary material for: Contribution of the efflux pump AcrAB-TolC to the tolerance of chlorhexidine and other biocides in Klebsiella spp
Source: J Med Microbiol. 2022 Mar 24;71(3):001496. doi: 10.1099/jmm.0.001496 (PMC9176267; doi:10.1099/jmm.0.001496)
Supplement: Supplementary material 1 [file jmm-71-1496-s001.pdf]

Supplementary Figures and Tables

Supplementary Table 1 – Bacterial strains used in this study. Acquired antibiotic resistance markers were generated using RES Finder.

Strain	ST	Aminoglycoside	Beta-Lactam	Fluoroquinolone	Phenicol	Tetracycline
NCTC 7427	86		*blaSHV-1*	*oqxAB*		
KPUK02	86		*blaSHV-28*	*oqxAB*		
6	268		*blaSHV-11*	*oqxAB*		
NCTC 13438	258	*aadA2*<br>*aph(3')-Ia*	*blaSHV-11*<br>*blaKPC-3*<br>*blaOXA-9*	*oqxAB,*<br>*gyrA* S83I<br>*parC* S80I	*catA1*	
46704	258	*aac(3)-Iva*<br>*aph(4)-Ia*<br>*aadA1*<br>*aph(3')-1a*<br>*aac(6')-Ib*	*blaSHV-12*<br>*blaKPC-2*<br>*blaOXA-9*<br>*blaTEM-1A*	*oqxAB,*<br>*aac(6')Ib-cr*<br>*gyrA* S83I<br>*parC* S80I		
CFI_131_KPC-2	258	*aac(6')-Ib*	*blaTEM-1A*<br>*blaKPC-2*<br>*blaOXA-9*	*oqxAB,*<br>*aac(6')Ib-cr*<br>*gyrA* S83I<br>*parC* S80I		
CFI_141_KPC-3	258	*aac(6')-Ib*<br>*aadA2*<br>*aph(3')-1a*	*blaSHV-11*<br>*blaKPC-3*	*oqxAB,*<br>*aac(6')Ib-cr*<br>*gyrA* S83I<br>*parC* S80I		
CFI_147_KPC-2	258	*aph(3'')-Ib*<br>*aph(6')-Id*<br>*aadA1*<br>*aadA2*<br>*aph(3')-1a*	*blaCMY-2,*<br>*blaSHV-12*<br>*blaKPC-2*<br>*blaOXA-10,*<br>*blaTEM-1A*<br>*blaOXA-9*	*oqxAB*<br>*gyrA* S83I<br>*parC* S80I	*catA1*<br>*cmlA1*<br>*floR*	
MKP103	258	*aadA2,*<br>*aph(3')-Ia*<br>*aac(6')-Ib-cr*	*blaSHV-11*<br>*blaTEM-1A*<br>*blaOXA-9*	*oqxAB*<br>*aac(6')Ib-cr*<br>*gyrA* S83I<br>*parC* S80I	*catA1*	
K. oxytoca<br>CFI_080_KPC-2	2		*blaOXY-2-8*<br>*blaKPC-2*<br>*blaOXA-9*<br>*blaTEM-1A*	*oqxAB*		

Supplementary Table 2

Strain	CIP	CHL	DOX	CAZ
M6 (No CHD)	0.125-0.25	4	4-8	0.5
M6 (CHD 4 mg/L)	≤0.06	≤0.5	≤0.5	0.125
M6 (CHD 8 mg/L)	≤0.06	≤0.5	≤0.5	0.125
MGH 78578 (No CHD)	2	512	128	>64
MGH 78578 (CHD 4 mg/L)	0.25	64	16	>64
MGH 78578 (CHD 8 mg/L)	0.25	32	8	64
M3 (No CHD)	0.125-0.25	16-32	2	>64
M3 (CHD 4 mg/L)	≤0.06	≤0.5	≤0.5	16
M3 (CHD 8 mg/L)	≤0.06	≤0.5	≤0.5	4

Supplementary Table 2 – MIC values in the presence of sub-lethal levels of chlorhexidine (CHD) for antibiotics known to be subject to efflux by AcrAB-TolC. All values are given as mg/L. Antibiotics used include ciprofloxacin (CIP), chloramphenicol (CHL), doxycycline (DOX) and ceftazidime (CAZ).

Supplementary Table 3 – MIC values against select biocides for MKP103 transposon mutants

Strain	Mutant	CHD	BAC	BEC	CPC	CTAB	DDAB	ALX	DQC	TRC	ETH	OCT	CET	HDPCM	NaDCC	VRK	PAD	H_2O_2	GLT
MKP103	-	64	32	64	8-16	64	16	2-4	256	2-4	6.25	4-8	0.004 - 0.007	16	125-250	0.5	0.04	0.005	0.39
KP02744	Δ*acrA*	8	16-32	64	8	64	8	4	32	1	6.25	2-4	0.002-0.004	8	125	0.5	0.04	0.005	0.39
KP02740	Δ*acrB*	4-8	16-32	64	8	32	8	4	16-32	1	6.25	4	0.001-0.004	4-8	62.5-125	0.5	0.04	0.01	0.39
KP02746	Δ*acrR*	64	32	64	16	128	16	4	256	2	6.25	4-8	0.015-0.03	16	250	0.5	0.04	0.005	0.39
KP09901	Δ*envR*	64	32	64	8-16	64	8	2	256	4	6.25	8	0.004 - 0.007	8	250	0.5	0.04	0.01	0.39
KP05254	Δ*marA*	64	32	64	8-16	64	16	4	256	4	6.25	4	0.004	8	250	0.5	0.04	0.01	0.39
KP05377	Δ*marR*	64	32	64	8-16	64	16	2	256	4	6.25	4	0.004	16	250	0.5	0.04	0.005	0.39
KP03202	Δ*ramA*	32	16	64	8	16	8	2	256	2	6.25	4	0.002	8	125-250	0.5	0.04	0.005	0.39
KP03197	Δ*ramR*	64	32	64	8-16	256	16	4	256	2	6.25	4	0.004-0.007	16	125-250	0.5	0.04	0.005	0.39
KP01732	Δ*rob*	64	32	64	8-16	64	8	2	256	4	6.25	4	0.004	16	125-250	0.5	0.04	0.01	0.39
KP06904	Δ*sdiA*	64	32	64	16	64	8	2	256	2	6.25	4	0.004 - 0.007	16	250	0.5	0.04	0.005	0.39
KP00770	Δ*soxR*	64	32	64	8	32	16	2	256	4	6.25	4	0.004	8	125-250	0.5	0.04	0.005	0.39

Supplementary Table 3 – Susceptibility of transposon mutants from *K. pneumoniae* MKP103 to selected biocides. All values given as mg/L except for ethanol (ETH (%)), cetrimide (CET (%)), Virkon (VRK (%)); peracetic acid (PAD (%)), hydrogen Peroxide (H_2O_2 (%)) and sodium hypochlorite (NaDCC (ppm)). Other biocides tested included alexidine dihydrochloride (ALX), dequalinium chloride hydrate (DQC), cetylpyridinium chloride (CPC), hexadecylpyridinium chloride monohydrate (HDPCM), chlorhexidine digluconate (CHD), cetyltrimethylammonium bromide (CTAB), didecyldimethylammonium bromide (DDAB), octenidine hydrochloride (OCT), benzalkonium

chloride (BAC), benzethonium chloride (BEC), triclosan (TRC) and glutaraldehyde (GLT). Values highlighted by light grey or dark grey shading indicate a 2-fold or >2-fold change in MIC, respectively, relative to w/t.

Supplementary Table 4 – MIC values against select antibiotics for MKP103 transposon mutants

Strain	Mutant	NOR	MXF	CIP	NAL	LVX	TOB	GEN	STP	AZI	CAZ	CTX	FOX	TGC	DOX
MKP103	-	>1024	512	>1024	>1024	512	128	8-16	128	>256	2-4	4	>64	8	64
KP02744	Δ*acrA*	1024	128	256	>1024	256	128	8-16	128	256	0.5	0.125	16	1-2	8
KP02740	Δ*acrB*	>1024	128	512	>1024	128	64-128	8	128	>256	1	1-2	64	0.5-1	16
KP02746	Δ*acrR*	>1024	512	>1024	>1024	512	128	16	128	>256	2	4	>64	8	64
KP09901	Δ*envR*	>1024	512	>1024	>1024	512	128	8-16	128	>256	2	2	>64	8	64
KP05254	Δ*marA*	>1024	512	>1024	>1024	512	128	8-16	128	>256	2	4	>64	8	64
KP05377	Δ*marR*	>1024	256	>1024	>1024	512	128	8-16	128	>256	2	4	>64	8	32-64
KP03202	Δ*ramA*	>1024	128	512	>1024	256	128	8-16	128	>256	1	1	64	1-2	8
KP03197	Δ*ramR*	>1024	256	>1024	>1024	512	128	8-16	128	>256	2	4	>64	8	64
KP01732	Δ*rob*	>1024	256	>1024	>1024	512	128	8-16	128	>256	2	4	>64	8-16	32

Supplementary Table 4 – Susceptibility of transposon mutants from *K. pneumoniae* MKP103 to selected antibiotics. MIC values are given as mg/L for the antibiotics norfloxacin (NOR), moxifloxacin (MXF), ciprofloxacin (CIP), naladixic acid (NAL), levofloxacin (LVX), tobramycin (TOB), gentamicin (GEN), streptomycin (STP), azithromycin (AZI), cefoxatin (FOX), ceftazidime (CAZ), cefotaxime (CTX), cefoxatin (FOX), tigecycline (TGC) and doxycycline (DOX). Values highlighted by light grey or dark grey shading indicate a 2-fold or >2-fold change in MIC respectively, relative to w/t.

Supplementary Table 5 – Mutations observed after adaptation to chlorhexidine

CHD adapted strain	Mutation	MGH 78578 equivalent	Function
MKP103	SmvR Q105STOP	KPN_01878	Regulator of MFS pump SmvA
	BamA L377Q	KPN_00190	Outer membrane protein assembly factor
KP03197 (Δ*ramR*)	*smvR* duplication of nucleotides 52-58	KPN_01878	Regulator of MFS pump SmvA
	RcnA E87D	KPN_00423	Efflux system for nickel and cobalt
KP03202 (Δ*ramA*)	*smvR* deletion of nucleotides 52-58	KPN_01878	Regulator of MFS pump SmvA
	in promoter of hypothetical protein	-	-
KP05925 (Δ*smvA*)	*sspA* deletion nucleotide 246	KPN_03628	Stringent starvation protein A
	mipA deletion nucleotide 471	KPN_01194	MltA-interacting protein

Supplementary Table 6 – Change in MIC of biocides after adaptation to chlorhexidine. All values given in mg/L except for cetrimide (CET (%)). Other biocides tested included cetylpyridinium chloride (CPC), hexadecylpyridinium chloride monohydrate (HDPCM), chlorhexidine digluconate (CHD), cetyltrimethylammonium bromide (CTAB), octenidine hydrochloride (OCT), benzalkonium chloride (BAC) and triclosan (TRC). Fold change against pre-adapted strain is indicated in brackets. For efflux pump status, ↑ indicates upregulated, ↓ indicates downregulated and b indicates basal level.

	CHD	CTAB	CPC	HDPCM	TRC	BAC	OCT	CET	Efflux Pump Status
MKP103	64-128	32	16-32	8-16	8	16	4	0.0015-0.003	↑*acrAB* b*smvA*
MKP103 CHD	512 (4-8)	64-128 (2-4)	32-64 (1-2)	64 (4-8)	8 (1)	16 (1)	4 (1)	0.007 (2-4)	↑*acrAB* ↑*smvA*
MKP103Δ*ramA*	32-64	16	8	8	4	8-16	2-4	0.0007-0.0015	↓*acrAB* b*smvA*
MKP103Δ*ramA*CHD	128-256 (4)	16-32 (1-2)	8-16 (1-2)	16 (2)	4 (1)	8 (1)	2-4 (1)	0.0015 (1)	↓*acrAB* ↑*smvA*
MKP103Δ*ramR*	64-128	32	16	8-16	8	8-16	4	0.0015-0.003	↑*acrAB* b*smvA*
MKP103Δ*ramR*CHD	256-512 (4)	64 (2)	32-64 (2-4)	32 (2-4)	8 (1)	8-16 (1)	4 (1)	0.007-0.015 (4)	↑*acrAB* ↑*smvA*
MKP103Δ*smvA*	16-32	16-32	8	8	8	16	2-4	0.0015	↑*acrAB* ↓*smvA*
MKP103Δ*smvA* CHD	64-128 (4)	16(1)	8 (1)	8 (1)	8-16 (1-2)	8-16 (1)	2-4 (1)	0.0015 (1)	↑*acrAB* ↓*smvA*

Supplementary Figure S1

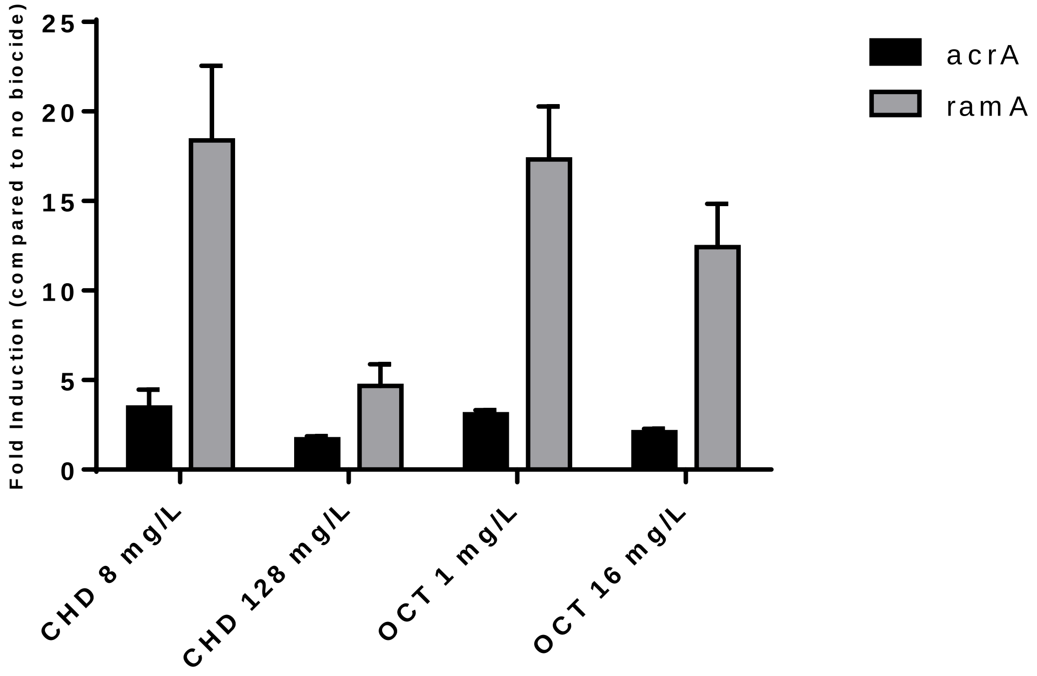

Figure S1 – Upregulation of *acrA* and *ramA* following challenge with chlorhexidine (CHD) or octenidine (OCT). Results are in triplicate and values are represented as the mean fold change, error bars are the SEM.
